# Retrospective, Landmark Analysis of Long-term Adult Morbidity Following Allogeneic HSCT for Inborn Errors of Immunity in Infancy and Childhood

**DOI:** 10.1007/s10875-022-01278-6

**Published:** 2022-05-17

**Authors:** James W. Day, Reem Elfeky, Bethany Nicholson, Rupert Goodman, Rachel Pearce, Thomas A. Fox, Austen Worth, Claire Booth, Paul Veys, Ben Carpenter, Rachael Hough, H. Bobby Gaspar, Penny Titman, Deborah Ridout, Sarita Workman, Fernando Hernandes, Kit Sandford, Arian Laurence, Mari Campbell, Siobhan O. Burns, Emma C. Morris

**Affiliations:** 1 Department of Immunology, Royal Free London Hospitals NHS Foundation Trust, London, UK; 2 Kings College London, London, UK; 3 University College London Hospitals NHS Foundation Trust, London, UK; 4 UCL Institute of Immunity & Transplantation, London, UK; 5 Great Ormond Street Hospital for Children, NHS Foundation Trust, London, UK; 6 UCL Great Ormond Street Institute of Child Health, London, UK; 7 Nepa, London, UK

**Keywords:** Very long-term outcome, allogeneic HSCT for IEI

## Abstract

**Purpose:**

Allogeneic hematopoietic stem cell transplant (HSCT) remains the treatment of choice for patients with inborn errors of immunity (IEI). There is little published medical outcome data assessing late medical complications following transition to adult care. We sought to document event-free survival (EFS) in transplanted IEI patients reaching adulthood and describe common late-onset medical complications and factors influencing EFS.

**Methods:**

In this landmark analysis, 83 adults surviving 5 years or more following prior HSCT in childhood for IEI were recruited. The primary endpoint was event-free survival, defined as time post-first HSCT to graft failure, graft rejection, chronic infection, life-threatening or recurrent infections, malignancy, significant autoimmune disease, moderate to severe GVHD or major organ dysfunction. All events occurring less than 5 years post-HSCT were excluded.

**Results:**

EFS was 51% for the whole cohort at a median of 20 years post HSCT. Multivariable analysis identified age at transplant and whole blood chimerism as independent predictors of long-term EFS. Year of HSCT, donor, conditioning intensity and underlying diagnosis had no significant impact on EFS. 59 events occurring beyond 5 years post-HSCT were documented in 37 patients (45% cohort). A total of 25 patients (30% cohort) experienced ongoing significant complications requiring active medical intervention at last follow-up.

**Conclusion:**

Although most patients achieved excellent, durable immune reconstitution with infrequent transplant-related complications, very late complications are common and associated with mixed chimerism post-HSCT. Early intervention to correct mixed chimerism may improve long-term outcomes and adult health following HSCT for IEI in childhood.

**Supplementary Information:**

The online version contains supplementary material available at 10.1007/s10875-022-01278-6.

## Introduction

Inborn errors of immunity (IEI) are rare, serious genetic disorders affecting innate and adaptive immunity. Clinical manifestations vary, but include infection, autoimmunity, inflammation and a predisposition to malignancy. Allogeneic hematopoietic stem cell transplantation (HSCT) has been performed as a curative therapy for many severe IEIs for over 50 years, now with excellent outcomes [1–4]. In severe combined immunodeficiency (SCID), HSCT is performed in very early childhood and overall survival rates of > 90% are typically reported. However, optimal timing of transplant remains controversial for a number of less severe IEI, but early transplant is increasingly advocated for certain disorders such as Wiskott–Aldrich syndrome (WAS) [5] and chronic granulomatous disease (CGD) if suitable donors are available [3, 6]. With increasing numbers of previously transplanted IEI patients now transferring to adult clinical care, detailed assessment of very long-term outcome in adulthood is only now assessable.

Post-transplant complications include engraftment failure, poor/inadequate immune reconstitution leading to increased susceptibility to infection and/or recurrence of IEI associated symptoms, graft versus host disease (GVHD) and death. Factors shown to influence early outcome post HSCT include underlying diagnosis, age at transplant, infection at time of transplant, conditioning regimen, donor type and HLA disparity [1, 7–10]. The use of reduced intensity conditioning (RIC) regimens, high-resolution HLA typing, earlier diagnosis and increasingly effective treatments for transplant-related complications have all contributed to improvements in survival over time [11–15]. Despite this progress, disease-related comorbidities and other transplant complications can result in long-term medical problems in transplant survivors. RIC regimens are associated with an increased risk of mixed chimerism (MC), which may undermine the durable reconstitution of normal, functional immunity. CD34^+^ stem cell top ups have been used for persistent cytopenias or incipient graft failure, but these do little to address MC. MC can only be reversed by a reduction in immune-suppression or donor lymphocyte infusions (DLI), both of which require extreme caution to prevent unacceptable GVHD risks [16, 17] and are not routinely used in pediatric non-malignant HSCT.

As the majority of IEI patients achieve good survival rates post-transplant, late and very late complications including malignancy, autoimmune disease, organ failure, infertility, growth, endocrine, respiratory, visual, and dental problems together with non-hematological disease-specific late complications are increasingly important [18–23].

Published long-term outcome data typically reports a median follow-up after HSCT of 3–11 years [1, 3, 4, 24], except for one large, single center study of transplanted SCID patients with a median of follow-up of 14 years (range 2–34 years) [25], demonstrating that persistent MC was associated with poor immune reconstitution and requirement for immunoglobulin replacement therapy (IRT). Elsewhere, a study of 74 infants with severe T cell immunodeficiency surviving at least five years post-HSCT were all still alive at last follow-up with good quality of life in the majority [21].

Here we report detailed medical complications occurring beyond five years post-transplant in 83 surviving patients with a median follow-up post-transplant of 20 years. Such very long-term outcome is essential to inform both service development and highlight pediatric peri- or post-transplant interventions which may be optimized further.

## Methods

### Study Design

Eligibility criteria for this retrospective study included (i) HSCT before the age of 16 years and (ii) 5 or more years survival following first transplant. We identified 212 IEI patients who were transplanted < 16 years at Great Ormond Street Hospital for Sick Children NHS Foundation Trust (GOSH) or University College London Hospitals NHS Foundation Trust (UCLH). Of these, 81 had died within five years of transplantation. Of the remaining 131 patients contacted, 83 consented to take part in our long-term psychological and medical outcome studies, while 48 declined to do so. All participating patients provided written informed consent as per institutional guidelines.

Data were collected from clinic letters, laboratory results, imaging and inpatient hospital records. Information on genetic/clinical diagnosis, HSCT characteristics (donor, conditioning, age at transplant, era of transplant), chimerism, immune reconstitution, GVHD, infectious complications, non-immunological medical outcomes and fertility was collected.

### Patient Demographics

The median age at last follow-up was 25 years (range: 17–39 years), with a median post-transplant follow-up duration of 20 years (range: 8–39 years; IQR: 7). Median age at transplant was three years (range: 0–18 years), 57 patients were male (69%) and 26 female (31%). Detailed patient demographics and transplant characteristics are shown in Table 1. IEIs were diagnosed using international criteria [26]. Underlying diagnoses were categorized into 3 groups: severe combined immunodeficiency (SCID) (*n* = 37); combined immunodeficiency (CID) (*n *= 33) and phagocyte disorders (*n* = 13), with details in Table 1. Median age at HSCT was significantly younger for SCID patients (5 m) when compared to CID (9 yr 8 m, *p* = *0.0001*) and PD (6 yr, *p* = *0.0001*) patients.

**Table 1:** Patient demographics and transplant characteristics

Patients (*n* = 83)	
Sex – no. (%)	
Male	57 (69%)
Female	26 (31%)
Median Age in years at last follow-up (range)	25 (17–39)
Median follow-up time in years (range)	20 (8–39)
PID diagnosis—no. (% total cohort)	
SCID group	37 (44.5%)
X-linked SCID	15 (18%)
ADA SCID	6 (7%)
Genetically undefined SCID	6 (7%)
Rag 1 SCID	4 (5%)
Rag2 Omenn SCID	3 (4%)
PNP SCID	1 (1%)
Artemis SCID	1 (1%)
JAK3 SCID	1 (1%)
CID group	33 (40%)
Genetically undefined CID	10 (12%)
WAS	8 (10%)
CD40L deficiency	4 (5%)
XLP	3 (4%)
DOCK8 deficiency	2 (2%)
APDS2	1 (1%)
X-L thrombocytopenia	1(1%)
RAG2 CID	1(1%)
CARD11	1(1%)
CTPS1	1(1%)
CHH	1(1%)
Phagocyte disorders group	13 (16%)
CGD	7 (8%)
Chediak Higashi syndrome	2 (2%)
LAD1 deficiency	2 (2%)
Undefined neutrophil disorder	2 (2%)
Donor (HLA mismatch) – no. (%)	
10/10 MRD	31 (37%)
Haplo	10 (12%)
MUD	36 (43%)
MMUD (1 or 2Ag MM)	6 (7%)
Conditioning Intensity– no. (%)	
Myeloablative (Bu/Cy; Flu/TBI)	32 (39%)
RIC (Flu/Mel; Flu/Bu; Flu/Treo)	38 (46%)
Unconditioned	13 (15%)
Age at transplant in years– no. (%)	
Under 1 year	30 (36%)
Between 1 and 4 years	17 (20%)
Over 5 years	36 (43%)
Median age at transplant for disease groups (range)	
SCID	5 m (1 day – 3 yr 3 m)
CID	9 yr 8 m (1 yr 1 m—15 yr 9 m)
PD	6 yr (1 yr 8 m – 14 yr 11 m)
Era of transplant – no. (%)	
Before 1996	19 (23%)
Between 1996 and 2001	26 (31%)
Between 2001 and 2006	27 (33%)
After 2006	11 (13%)

Abbreviations: *ADA* adenosine deaminase; *PNP* purine nucleoside phosphorylase; *SCID* severe combined immunodeficiency; *CHH* cartilage-hair hypoplasia; *APDS* activated PI3K delta syndrome; *XLP* X-linked lymphoproliferative disease; *WAS* Wiskott–Aldrich syndrome; *CGD* chronic granulomatous disease; *LAD1 deficiency* Leukocyte adhesion deficiency type 1; *MRD* matched related donor; *Haplo* haploidentical; *MUD* Matched unrelated donor; *MMUD* mismatched unrelated donor; *RIC* Reduced intensity conditioning. See text for details of conditioning regimens

Demographic data of long-term survivors who declined to take part (*n* = 39, 81% of the 42 not reported) demonstrates the study population can be considered broadly representative of all long-term survivors. There was no significant difference between the distribution of underlying diagnoses (*p* = 0.834), median length of follow-up post HSCT (*p* = 0.127), age at HSCT (*p* = 0.034) or era of HSCT (*p* = 0.063) between the study patients and the patients not consenting to the study. Further, OS was 98% for those in the study (*n* = 83) and 96% for non-consented, *p* = 0.437. Data not shown.

### Transplant Characteristics

Data on donor status, conditioning regimen intensity, age at transplant and era of transplant are also shown in Table 1. 36 patients received stem cells from 10/10 HLA-matched unrelated donors (MUD), 31 patients had 10/10 HLA-matched related donors (MRD), 10 transplant recipients had haploidentical donors (Haplo) and six patients had either one or two Ag-mismatched unrelated donors (MMUD). Most patients (*n* = 69; 83%) received conditioning chemotherapy prior to infusion of allogeneic stem cells. 38 patients (46%) received various reduced intensity conditioning (RIC) regimens, in line with the era of transplant including fludarabine and melphalan (Flu/Mel), fludarabine and busulphan (Flu/Bu) or fludarabine and treosulphan (Flu/Treo). Of the patients receiving myeloablative conditioning (MAC), 31 received busulfan and cyclophosphamide (Bu/Cy) and one received fludarabine and total body irradiation (TBI). A total of 30 patients (36%) were transplanted before one year of age, 17 patients between one and four years, and 36 at the age of five years or older (only two patients were transplanted above the age of 15). Due to the time required post-HSCT for patients to transition to adult care and be eligible for recruitment into this study those under the age of 5 yrs at HSCT where HSCT was performed *after* 2006 would not have reached the age required to be included in this study. To assess the potential impact of change in transplant practice over time, our patients were also grouped by transplant era. 19 patients received their first HSCT before 1996, 26 between 1996 and 2001, 27 between 2001 and 2006, and a further 11 after 2006. Reflecting changes in transplant practice over time, no RIC transplants were performed pre 1996 an no unconditioned transplants were performed after 2006.

Seven patients (8.4% of the study cohort) had received a second transplant at an average of 1.7 years post HSCT, but only 3 of these occurred beyond 5 years post initial HSCT and were included as events.

### Primary Outcome Measures

#### Event-Free Survival

The primary endpoint for our study was event-free survival (EFS). Date of last follow-up was the date of last clinical review. EFS was defined as the time from first transplant to an event. In this landmark analysis, *only* events occurring at or after 5 years post 1^st^ HSCT were included for the EFS analysis. Events were defined as: the need for graft-related second intervention (conditioned allograft, DLI, CD34 + top-up), chronic viral infection, life-threatening infection, recurrent acute infection requiring antibiotic treatment, de novo or relapsed malignancy, significant autoimmune disease requiring systemic immunosuppression and/or medical treatment, moderate to severe GVHD [27], major organ dysfunction and death. To describe the burden of late medical events seen in this cohort of long-term survivors reaching adulthood we have included events related to both the underlying disease and the HSCT.

Chronic viral infection included debilitating, extensive and intractable viral warts, recurrent herpetic outbreaks (> 3 episodes/year) requiring systemic treatment, and chronic (> 1 year) EBV viremias (> 25,000 copies/ml) with or without treatment requiring intensive monitoring. Recurrent acute infections were defined as three or more infections per year, each requiring oral or intravenous antibiotics. To be considered an event, organ dysfunction had to be chronic (> 1 year) and of at least moderate severity (according to NCI CTCAE criteria). The number of patients who were free of any major complications (listed above) at last follow-up was also documented. See Supplementary Data Table 1 for detailed description of all events in our cohort.

### Secondary Outcome Measures

#### Chimerism Analysis

Peripheral blood chimerism analysis was performed using validated techniques (fluorescence in situ hybridization or polymerase chain reaction of short tandem repeats). Lineage-specific chimerism was performed on peripheral blood mononuclear cells (PBMCs) and T cell (CD3^+^), B cell (CD19^+^), and granulocyte (CD15^+^) fractions. Lineage-specific chimerism results were reported as ≥ 95% donor; ‘mixed’ 50–94% donor; ‘very mixed’ 5–49% donor; or ‘recipient’ < 5% donor.

#### Immune Reconstitution and Vaccine Responses

Lymphocyte subset analysis, T cell proliferation and immunoglobulin levels were performed on all patients. Enzyme-linked immunosorbent assays (ELISAs) were used to detect serological responses to *pneumococcus,* with measurement of serotype-specific anti-pneumococcal IgG responses available in a proportion of patients. T cell proliferation was measured after stimulation with phytohemagglutinin (PHA), anti-CD3 and anti-CD3/CD28 antibodies using validated diagnostic assays. The stimulation index (SI) was calculated by dividing the proliferation after stimulation with proliferation of unstimulated cells (background). The ranges for normal, impaired and absent were determined based on healthy control values.

### Statistical Analysis

OS and EFS were calculated from 5 years post-transplant in this landmark analysis. Events occurring prior to 5 years were not considered in these analyses. Probability of EFS was calculated using the Kaplan–Meier method with Stata 16 (StataCorp. 2019. Stata Statistical Software: Release 16. College Station, TX: StataCorp LLC.) and comparisons of EFS curves were made using a multi-state Cox model, with event-free, event and death as the states and independent transitions between the states. Transplant characteristics, chimerism and immune reconstitution, and the occurrence of medical events were assessed in univariate Cox models, and all factors with p < 0.2 were included in multivariable models. Factors not significant in the multivariable model were then excluded. The proportional hazards assumption of the Cox model was checked by visual inspection of the proportional hazard (log–log) plots (for categorical variables) and by the Schoenfeld residuals test. The only univariate models for which the proportionality test failed were year of transplant, either by five-year interval (*p* = 0.012) or as a continuous variable (*p* = 0.011). Likewise, the multivariable model showed no evidence for violation of proportionality (*p* = 0.34). For time varying factors (such as chimerism) the patients’ time after transplant was further split at each time point when chimerism was tested. Multivariable models were constructed using all terms with > 90% data completeness and with *p* < 0.2 in univariate models.

Lymphocyte subset counts (Table 4) for adults are presented raw, but those for pediatric patients (while they were < 16 years) were normalized to the median of the age-adjusted reference range. The reference ranges were similarly normalized and averaged over the age range. The reference median is thus 1.0 and the reference range displayed is the mean ratio of the reference limits.

## Results

### Event-Free Survival (EFS)

**Fig. 1:**
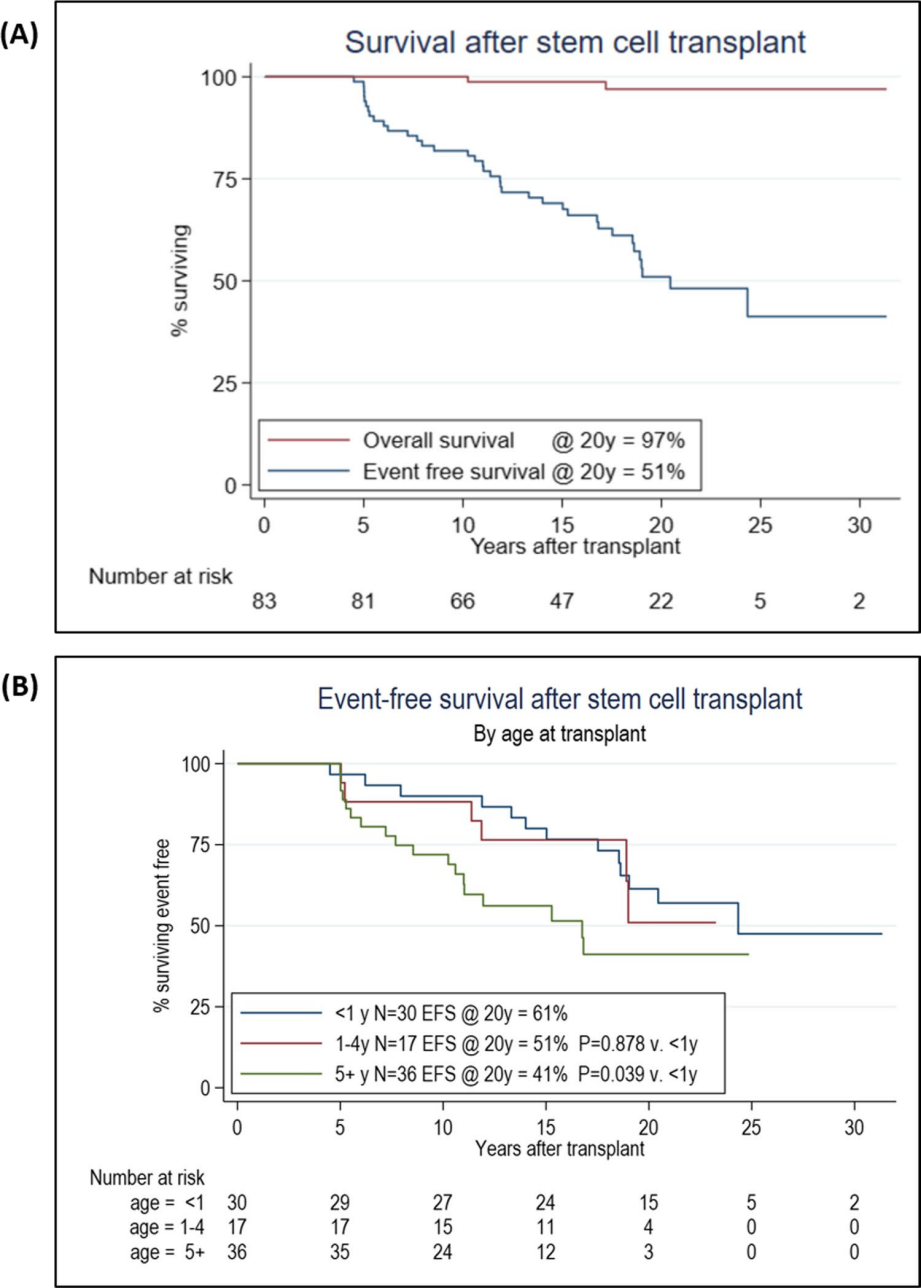
Overall and event-free survival. **A.** Overall survival (OS) was 97% and event-free survival (EFS) was 51% at 20 years post-transplant for our cohort (*n* = 83). **B.** Impact on EFS of age at transplant. EFS at 20 years post-transplant was 61% in patients less than 1 year, 51% in those aged 1 to 4 years, and 41% in those 5 years or older at transplant

At 20 years post-transplant, the EFS was 51% in the overall cohort (Fig. 1A); 54% in the SCID group, 50% in the CID group and 54% in the phagocyte disorder group (*p* = 0.35, Table 2). Univariate analysis identified age at transplant and CD4 count at five years post-transplant as significantly impacting 20 yr EFS (Table 2), although only 2 patients had CD4 counts < 0.2 × 109/l at 5 years post-transplant. Patients transplanted at age < 1 yr, 1-4yrs and ≥ 5yrs had subsequent 20 yr EFS of 60%, 51% and 41% respectively (*p* = 0.04 for age ≥ 5yrs compared to < 1 yr at HSCT, Fig. 1B and Table 2).

**Table 2:** Statistical analysis of event-free survival

Factor	Levels	N	Pts with events	EFS @ 20y (95% CI)	Univariate		Multivariable	
					HR (95% CI)	*P* value	HR (95% CI)	*P* value
Overall		83	37	51% (39—63)				
Diagnosis	SCID	37	18	54% (35—69)	1.0			
	CID	33	13	50% (28—69)	1.22 (0.59 – 2.52)	0.35		
	PD	13	6	54% (25—76)	1.55 (0.60 – 3.98)			
Year of HSCT	< 1996	19	11	95% (68—99)*				
	1996–2000	27	13	70% (49—84)*				
	2001–2005	27	8	93% (74—98)*				
	2006–2011	10	5	60% (25—83)*				
	continuous				1.02 (0.96 – 1.08)	0.52		
Conditioning	None	13	8	44% (17—68)	1.0			
	MAC	32	13	59% (39—74)	0.66 (0.27 – 1.60)	0.279		
	RIC	38	16	51% (32—68)	1.05 (0.44 – 1.52)			
Age at 1^st^ transplant	< 1 yr	30	13	61% (41—77)	1.0		1.15 (1.05 – 1.25)	0.002
	1-4 yr	17	6	51% (18—77)	1.08 (0.41 – 2.88)	0.88*		
	5 + yr	36	18	41% (22—59)	2.18 (1.04 – 4.56)	0.04*		
Donor	MRD	31	14	54% (34 -71)	1.0	0.86		
	Haplo	10	6	48% (16—74)	1.21 (0.47 – 3.16)			
	MUD	36	17	43% (23—61)	1.26 (0.62 – 2.57)			
	MMUD	6	0	100%	0			
CD4 count at 1 yr	< 0.2 ≥ 0.2	10 73	4 33	60% (25—83) 51% (38—63)				
	Continuous				0.63 (0.22 – 1.79)	0.42		
CD4 count at 5 yr ^§^	< 0.2 ≥ 0.2	2 81	2 35	0% 52% (39—64)				
	Continuous				0.10 (0.02 – 0.61)	0.01		
WB Chim at 1 yr	≥ 95%	47	19	50% (32—66)				
	< 95%	23	12	55% (33—73)				
	Continuous				0.95 (0.43 – 2.08)	0.90		
WB Chim at 5 yr ^§^	≥ 95%	35	11	58% (34—76)				
	< 95%	34	16	53% (34—69)				
	Continuous				0.35 (0.10 – 1.29)	0.12	0.12 (0.03 – 0.55)	0.006
On IRT	Yes	6	3	50% (11—80)	1.75	0.36		
	No	77	34	62% (50—72)	(0.53 – 5.79)			

All variables included in a backwards selection time-dependent Cox regression model for survival from 5y post-transplant, with *p* < 0.05 for inclusion and *p* > 0.20 for removal. Forward selection produces the same model. *pairwise comparison with age < 1 yr at HSCT. Analysis of CD4 counts based on normalized values

Abbreviations: *SCID* severe combined immunodeficiency; *CID* Combined immunodeficiency; *PD* phagocytic defect; *CI* Confidence interval; *HR* Hazard ratio

^*^at 10y for comparability; § EFS from 5y; *Chim* chimerism; *Cont* Continuous; *MRD* matched related donor; *MUD* matched unrelated donor; *MMUD* mismatched unrelated donor; *Haplo* Haploidentical; *RIC* Reduced intensity conditioning; *MAC* myeloablative conditioning; *IRT* immunoglobulin replacement therapy

However, year of HSCT, donor, conditioning intensity, underlying diagnosis, whole blood chimerism at 1 year and lineage-specific chimerism had no significant impact on EFS by univariate analysis (Table 2).

Multivariable analysis confirmed only age at transplant (*p* = 0.002, Table 2) and whole blood chimerism, at the fixed time point of five years post HSCT (*p* = 0.006, Table 2) as independently significant in predicting EFS beyond 5 years post-transplant. However, when analysis was performed in specific IEI subgroups, the impact of WB chimerism at 5 years on subsequent EFS was only significant for the SCID patients (Table 3). Within the SCID group, both age at HSCT and WB chimerism at 5 yrs influenced EFS (*p* = 0.01, *p* = 0.03, respectively). However, only age at HSCT was found to be significant for EFS in the PD group (*p *= 0.03), Table 3.

**Table 3:** Factors significantly impacting EFS within different IEI subgroups on multivariable analysis

IEI Subgroup	Factor	P value	HR
SCID	Age at HSCT	0.01	4.728 (1.55 – 14.44)
	WB chimerism at 5 yr (continuous)	0.03	0.016 (0.0003 – 0.682)
CID	WB chimerism at 5 yr (continuous)	0.07	0.155 (0.021 – 1.171)
PD	Age at HSCT	0.03	1.334 (1.029 – 1.728)

Abbreviations: *SCID* severe combined immunodeficiency; *CID* Combined immunodeficiency; *PD* phagocytic defect; *WB* whole blood; *IEI* inborn error of immunity; *HR* Hazard ratio

A total of 59 significant medical events were documented in 45% of the cohort (37 patients) (Supplementary Table 1). The most frequent events were chronic viral infection (*n* = 14), followed by recurrent/severe acute infection (*n* = 14), autoimmune disease (*n* = 10), moderate/severe organ dysfunction (*n* = 12), malignancy (*n* = 2), graft related secondary intervention (*n* = 3), moderate/severe GVHD (*n* = 2) and death (*n* = 2). All clinical events are described in Supplementary Table 1.

Two patients died after enrollment in the study at 33 and 10 years post-transplant, resulting in an overall survival in our cohort of 97% at 20 years (Fig. 1A). Cause of death included hepato-pulmonary syndrome secondary to hepatic nodular regenerative hyperplasia and bronchiectasis in the context of failed B cell immune reconstitution in a patient transplanted for SCID, and sudden cardiac death in a patient transplanted for X-CGD (P3 and P24, respectively, Supplementary Table 1).

Overall, 69% (*n* = 58) of patients were event-free at their last follow-up appointment (at a median of 20 years post-transplant), including those who had never had an event during the study period (*n* = 46) and those in whom prior events had resolved and no new events had occurred in the last 12 months of follow-up (*n* = 12). A total of 25 patients (30% of the cohort) were experiencing ongoing significant complications, including two patients who died of these, as discussed above. Only 11 (13%) patients had more than one clinically significant event.

### Infection

#### Late Bacterial and Fungal Infections

13 patients developed 14 infectious complications at a median onset of 15.9 years post-HSCT (range: 5–27 years) (see Supplementary Table 1). These included recurrent urinary tract and perineal skin infections (UTI) (P2); recurrent lower respiratory tract infections (LRTI) (P3, P17, P21, P22, P28); other infections requiring prolonged antibiotic and/or anti-fungal therapy, including TB (P5) and persistent fungal nail infection (P8). Five additional patients required hospital admission for bacterial meningitis (P32); bacterial pneumonia with subsequent orbital cellulitis (P18); bacterial pneumonia (P35); gastroenteritis (P31); and vocal fold candidiasis (P34). 12 of the 13 patients suffering late infectious complications had been transplanted for either SCID or CID. One patient had a diagnosis of Chediak Higashi syndrome.

#### Chronic Viral Infections

A total of 14 chronic (≥ 1 year duration) viral infection events were documented in 12 patients (14% of study cohort) with onset beyond five years post-transplant. 10 patients had severe (extensive and debilitating) HPV-associated viral warts; all these patients received HSCT for correction of SCID. All nine patients with gamma chain or JAK3 SCID developed warts at a median of 15.7 years post-HSCT (range: 8–20 years) and have been refractory to topical treatments and surgical intervention. Three patients had chronic EBV viraemia requiring intensive monitoring but no intervention (P1, P16, and P19); with one also developing severe HSV-associated recurrent genital herpes (P1). No patients transplanted for phagocyte disorders developed chronic viral infections post-transplant.

### Chronic Graft-Versus-Host Disease (GVHD)

Long-term chronic GVHD requiring systemic therapy was very rare, affecting two patients. While six patients (7% total cohort) suffered from chronic GVHD beyond five years post-transplant, only two had moderate/severe requiring systemic treatment and defined as events, one SCID patient after a CD34 + top-up and the other following RIC MRD allograft for XLP (P15 and P20). Four patients had limited chronic skin GVHD at last assessment, requiring topical corticosteroids only and were not included as significant events.

### Late Development of Autoimmune Disease

Nine patients (11% of total cohort) developed 10 de novo autoimmune events at a median of 10.5 years post-HSCT at a range of 3–17 years (P13 developed juvenile idiopathic arthritis, JIA at three years post-HSCT that persisted beyond five years post-HSCT). These diagnoses included autoimmune joint disease (*n* = 3), type 1 diabetes mellitus (*n* = 1), autoimmune hemolytic anemia (*n* = 2), transverse myelitis (*n* = 1), Guillain–Barre syndrome (*n* = 1), keratitis (*n* = 1) and uveitis (*n* = 1). Autoimmune diseases were distributed across the IEI diagnostic groups. No association between autoimmune disease and peripheral blood chimerism was observed. Two (2%) patients had documented secondary hypothyroidism (one with MAC and the other with RIC) without auto antibodies.

### Late Hepatobiliary, Renal and Respiratory Complications

Three patients (4% of the cohort) developed late onset moderate/severe renal or hepatic sequelae (chronic kidney disease (P13, P26) and liver failure (P3)). P13 received an unconditioned MRD infusion for genetically undefined SCID, developed JIA at three years post-HSCT requiring prolonged systemic immunosuppression and subsequently developed CKD-stage 3A at five years post-HSCT, presumed secondary to medications. P26, who received a RIC Fu/Bu/Alemtuzumab MUD allograft for X-CGD, developed CKD-stage 3A with hypertension and proteinuria at 7 years post-HSCT controlled with Ramipril, and related to a pre-HSCT vascular insult. P3 received a conditioned Haplo for homozygous RAG2 Omenn syndrome. She had a CD34 + top-up at 14 years post-HSCT for poor B cell recovery and absent myeloid engraftment associated with recurrent LRTI and bronchiectasis despite adequate IRT. She died of liver failure secondary to nodular regenerative hyperplasia (NRH) and hepato-pulmonary syndrome.

Five patients were diagnosed with chronic lung disease beyond five years post-HSCT: four cases of bronchiectasis and one case of bronchiolitis obliterans. Three patients with bronchiectasis were immunoglobulin dependent at last follow-up in the absence of B cell engraftment: P3 (RAG2 Omenn), P11 (Gamma chain SCID) and P19 (WAS). The fourth patient, P28 (genetically undefined SCID) had recurrent lower respiratory tract infections (LRTI) despite 100% engraftment across all lineages, with initial suboptimal pneumococcal vaccine responses, corrected by re-vaccination.

P27 developed focal bronchiolitis obliterans at five years post RIC MUD allograft for X-CGD. He had a whole blood chimerism level of > 95% at last follow-up and good immune reconstitution with adequate response to tetanus and pneumococcal vaccines. There was no evidence of pulmonary GVHD in our study population.

### Graft-related Subsequent Interventions beyond 5 years Post-HSCT

Three patients received a CD34 + top-up at a median of 17 years post-HSCT (range: 14–19 years) either to correct persistent cytopenias despite full donor chimerism (P37) or to correct persistent mixed chimerism (P3, P15). Two had myeloablative transplants (P3 and P15, respectively) and one received an unconditioned MSD infusion (P37). No impact on B cell engraftment was observed (see Supplementary Table 1).

### Late Malignancy

Two (2%) patients developed late malignancies including squamous cell carcinoma at 25 years (P15) and recurrent basal cell and squamous cell carcinoma at four and six years post-HSCT (P26), respectively. P15 (gamma chain SCID) also had chronic GVHD causing alopecia totalis and recalcitrant warts (HPV serotypes 17, 27, and 18) despite good immune reconstitution. His SCC (scalp) was positive for HPV18. P26 (X-CGD) was previously reported by Unni et al., 2018 and despite achieving 100% full donor chimerism required prolonged voriconazole for prophylaxis of CGD-associated fungal infections. He initially developed pre-malignant actinic keratosis and subsequently developed basal cell carcinoma of the ear and recurrent SCC of the lower leg at four years and then six years post-HSCT. He had chronic HPV-associated viral warts as well as chronic skin GVHD. A further two patients developed benign tumors: tubulo-papillary adenoma of the gallbladder at 17 years post-HSCT (P21) and a schwannoma of the lower jaw (P18) at 18 years post-HSCT.

### Chimerism

#### Whole Blood Chimerism

Chimerism results at last follow-up were available in 80 (96%) of our 83 patient cohort. Over half (43 patients, 54%) had achieved durable > 95% whole blood donor chimerism at last assessment. The median time from transplant to last chimerism analysis was 15 years (range: 1–33 years). 29% of SCID patients (*n* = 34) had ≥ 95% donor chimerism compared to 76% in the CID group (*n* = 33) and 62% in the phagocyte disorders group (*n* = 13) (*p* = 0.001, data not shown). The failure to achieve durable full donor chimerism in the majority of transplanted SCID patients is likely related to impaired stem cell engraftment in the 36% of SCID patients who underwent unconditioned transplants.

**Fig. 2:**
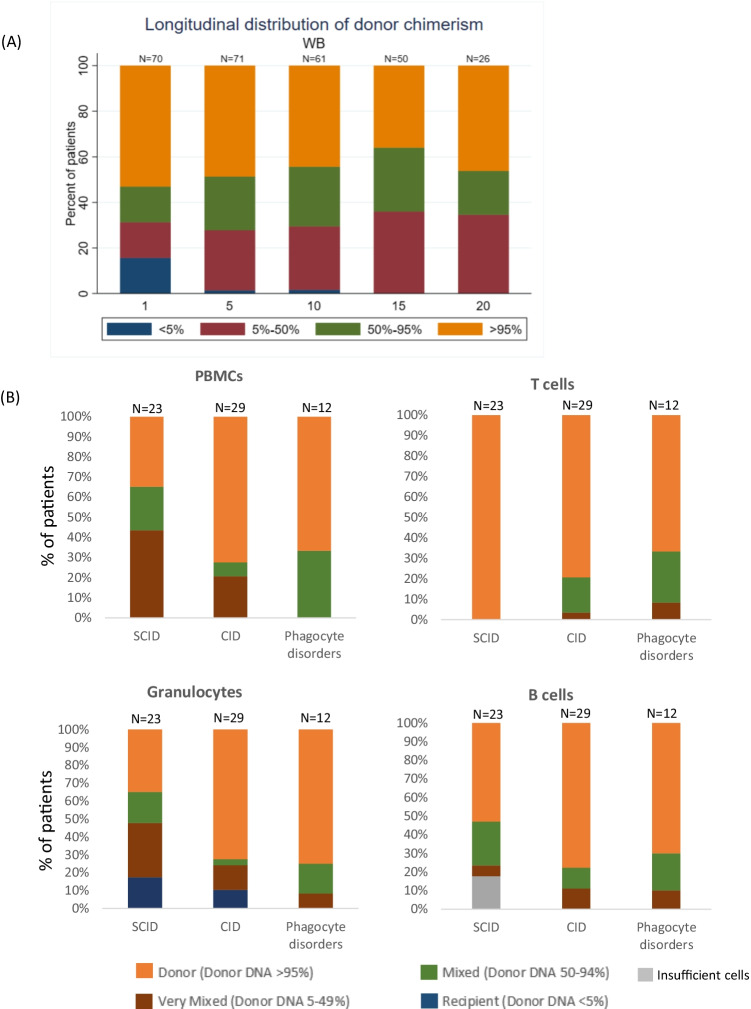
Post-transplant peripheral blood chimerism. **A.** Longitudinal distribution of whole blood chimerism at 1, 5, 10, 15 and 20 years post-transplant (*n* = 71). **B.** Lineage-specific peripheral blood chimerism at last follow-up (median 15 years post-transplant) for 62 patients (*n* = 23 SCID; *n* = 29 CID; and *n* = 12 PD). PBMC (upper left panel) shows 35% of SCID patients had more than 95% engraftment compared to 70% and 72% of CID and 67% of phagocyte disorder sub-groups respectively. T cell chimerism (upper right panel) shows that 100% of SCID patients had > 95% T cell engraftment compared to 79% and 67% of CID and phagocyte disorder sub-groups. Granulocyte engraftment (lower left panel) shows that 35% of SCID patients had > 95% engraftment, 72% CID and 75% phagocyte disorders. B cell engraftment (lower right panel) shows that 53% SCID patients had > 95% engraftment, 78% CID and 70% phagocyte disorders. Patients with FISH analysis only were excluded from this analysis

Longitudinal distribution of whole blood chimerism analysis at 5, 10, 15 and 20 years post-transplant was available for 71 patients and are shown in Fig. 2A.

#### Lineage-specific Chimerism

Lineage-specific chimerism at last follow-up was available in 62 patients and shown in Fig. 2B. Eight of the nine SCID patients who received unconditioned transplants and for whom lineage-specific chimerism was available, demonstrated full donor chimerism in the T cell fraction only.

### Immune Reconstitution

#### Immunoglobulins

At last follow-up, 88% of patients were no longer on IRT and had median IgG, IgA and IgM concentrations of 10.8, 1.0 and 1.75 g/L, respectively (Table 4A). The 10 (12%) patients requiring IRT at their last follow-up had been transplanted for SCID or CID. Three of these patients had unconditioned transplants and five had reduced intensity conditioning.

**Table 4:** Immune reconstitution at last follow-up

(A)						
**Immunoglobulins** Median (range), g/L (Normal ranges for adults: IgG 7.0–16.0; IgA 0.7–4.0; IgM 0.4–2.3 g/L)						
**Immunoglobulin replacement therapy** (No of patients)	**IgG**		**IgA**		**IgM**	
Off IRT at last f/u (n = 73)	10.8 (5.6–23.4)		1.0 (0.3–6.8)		1.75 (0.1–6.6)	
On IRT at last f/u (n = 10)	12.75 (9.1–18.1)		0.2 (0.1–1.2)		0.95 (0.1–4.1)	
(B)						
**Lymphocyte subsets** Median (range), × 10^9^/L (Normal ranges for adults: Lymphocytes 1.0–2.8; CD3 0.7–2.1; CD4 0.3–1.4; CD8 0.2–0.9; CD19 0.1–0.5; CD16CD56 0.09–0.6 × 10^9^/L)						
	**Total (n = 67)**	**CD3** **(n = 66)**	**CD4** **(n = 66)**	**CD8** **(n = 66)**	**CD19 (n = 63)**	**CD16 CD56 (n = 63)**
**SCID**	1.80 (0.96–3.32)	1.39 (0.78–2.51)	0.6 (0.27–1.46)	0.55 (0.21–1.24)	0.22 (0.01–0.65)	0.11 (0.02–0.64)
**CID**	1.6 (0.64–3.00)	1.16 (0.42–2.04)	0.59 (0.22–1.23)	0.47 (0.15–0.89)	0.19 (0–0.69)	0.15 (0.04–0.77)
**PD**	1.53 (0.84–2.75)	1.10 (0.51–2.05)	0.56 (0.28–1.29)	0.47 (0.15–1.21)	0.23 (0.15–0.54)	0.15 (0.27–0.05)
(C)						
**T cell proliferation** (Stimulation Index, SI)*						
	**PHA (n = 50)**		**CD3 (n = 47)**		**CD3/CD28 (n = 46)**	
Absent*	0		6		5	
Impaired*	2		5		3	
Normal*	48		39		42	
CPM Median (range)						
Patients Controls	415 (83–3419) 464 (104–1999)		63 (0.4–646) 119 (1–466)		193 (1–1078) 280 (11–1263)	
(D)						
**Pneumococcal CP Ab Response** No of pts (% tested)	**Pneumococcal serotype-specific responses** No of pts (% of tested)					
> 50 mg/L	≥ 7/13 protective (> 0.35 ug/ml)			≤ 6/13 protective (< 0.35 ug/ml)		
21 (72%)	39 (71%)			16 (29%)		

^*^PHA/CD3/CD3CD28: Absent SI ≤ 5/ ≤ 1/ ≤ 1; Impaired SI 5–99/2–20/2–50; Normal SI ≥ 100/ ≥ 20/ ≥ 50. Raw data for lymphocyte counts shown

*SCID* severe combined immune deficiency; *CID* combined immune deficiency; *PD* phagocytic disorder; *PHA* phytohemagglutinin; *CP* capsular polysaccharide (antigens)

#### Lymphocyte Subsets

Lymphocyte subset analysis was available in 63 patients from their last clinical assessment at a median time of 19.5 years post-transplant. Details of immune reconstitution are shown in Table 4B. Median lymphocyte counts were in the normal range for CD3, CD4, CD8, CD19 and CD16CD56 cell subsets.

#### T cell Proliferation

T cell proliferation results were available in 50 patients from their last clinical review. The vast majority of patients tested had normal T cell proliferation responses to PHA, CD3 and CD3/28. In the whole cohort, the median phytohemagglutinin (PHA) stimulation index (SI) was 415 (range 83–3419), median CD3 SI was 63 (range 0.4–646) and the median CD3/CD28 SI was 193 (range 1–1078) (Table 4C).

#### Vaccine Responses

Overall, 72% of patients had a pneumococcal CP antibody response above 50 mg/L. 71% of patients had a protective antibody response in ≥ 7/13 pneumococcal serotypes whilst 29% had protective levels in ≤ 6/13 (Table 4D). Time from last pneumococcal vaccination was not available.

## Discussion

This study is the first to examine the *very* long-term impact on adult health of HSCT in infancy or childhood for patients with IEI. We describe the medical outcomes for 83 patients who had survived five years or more post-transplant, thus excluding TRM deaths and early complications, with a median follow-up of 20 years (8–39 years). Despite excellent long-term survival, this study has identified factors associated with significantly worse EFS and ongoing medical complications after transition to adult care.

In our cohort, 45% of patients experienced one or more clinically significant medical event/s beyond five years post-transplant and 30% had ongoing complications at their last follow-up visit (median of 20 years post-transplant). Neither the underlying disease group, donor source or era of transplant influenced EFS in this study. As with other published studies, age at transplant (specifically < 1 yr compared to ≥ 5yrs) significantly impacted very late EFS (*p* = 0.04), with a better outcome for patients transplanted early.

On multivariable analysis, whole blood chimerism at 5 years post-HSCT (as continuous variable) influenced late EFS, suggesting that chimerism results can be an accurate predictor of long-term outcomes. However, when analyzed within IEI subgroups, whole blood chimerism remained significant for EFS only in SCID patients. There is little published data on the very late impact of mixed chimerism in non-SCID IEI and our study suggests this is less important in the non-SCID group. In HSCT for malignant disease chimerism is a predictor for overall survival and disease relapse [28, 29] as persistent mixed chimerism may be a surrogate for immune tolerance and lack of graft-versus-tumor effects. However, in non-malignant disease, and particularly IEI, the significance of mixed multi-lineage chimerism is complex and relates to disease pathogenesis. The presence of non-functional or dysfunctional recipient-derived immune cells long-term may generate an only partially corrected clinical phenotype and patients with full donor chimerism were more likely to remain free of complications long-term. Our results highlight the importance of monitoring chimerism during longer term follow-up, as in some patient groups (e.g., SCID), persistent mixed chimerism predicts for poorer long-term EFS. Larger studies in non-SCID IEI are required to address the impact of persistent mixed chimerism on long-term outcomes.

The observed rate of ongoing chronic GVHD in our study was very low, despite the majority of our patients receiving either MUD, MMUD or Haploidentical transplants, reflecting the use of serotherapy for TCD in a large number of our patients, the low age at transplant and exclusion of events occurring in the first 5 years post-transplant. The 10% rate of autoimmune disease we observed was similar to that previously reported [21, 25] and these events can arise de novo many years after HSCT. We did not find an association between late autoimmunity and mixed chimerism, which may have been due to insufficient patient numbers overall or in specific disease groups. Late isolated bacterial and fungal infections were seen, but persistent viral infections were the most common infectious long-term complication. In particular, persistent or recurrence of warts at very late timepoints post-transplant has been noted by others, with the highest risk in patients transplanted for X-SCID [25].

The known increased lifetime risk of developing a second malignancy following HSCT can be related to conditioning chemotherapy, GVHD, EBV infection, age at transplant and persistent immunodeficiency. 2% of our cohort developed a second malignancy, in keeping with previously described rates [30]. All three malignancies reported in our cohort were skin malignancies suggesting a role for dermatology follow-up.

Our study has a number of limitations. Firstly, some patients surviving childhood HSCT for IEI were lost to follow-up and not included in this study. This in part reflects earlier practice prior to establishment of a more robust transition service between the centers. As a result, we may have un-knowingly excluded patients without on-going medical needs. However, as we were unable to identify any significant differences between our study group and survivors who declined to take part, we believe the patients described are representative. Additionally, most patients had HSCT prior to 2006 and thus more recent advances in the management of IEI before and around HSCT may not be accounted for. Detailed chest imaging was often unavailable pre-HSCT and therefore we cannot exclude that bronchiectasis preceded HSCT in some patients. We have not reported the quality of life and psychological outcome of our cohort here and this is the subject of a separate manuscript.

We have shown that very long-term survivors of stem cell transplantation for IEI have a high rate of clinically significant medical events which can occur many years post-transplant. Patients with persistent mixed chimerism are more likely to suffer post-transplant complications and this group of patients may benefit from earlier attempts at reversing mixed chimerism by the judicious use of DLI. The recent introduction of targeted conditioning in pediatric transplantation is predicted to improve both immune reconstitution and donor chimerism with an eventual improvement in late outcomes [31, 32].

Nevertheless, the prevalence of ongoing medical issues related to both the original IEI and HSCT sequelae underlines the need for robust transition services leading to life-long follow-up. Long-term care for this group of patients should include input from multidisciplinary teams with both IEI and HSCT expertise. Detailed transfer of information about both the underlying IEI and the HSCT procedure is vital to provide optimal individualized care, and this is a particular challenge where pediatric immunology and transplant care have occurred in different locations. We have addressed the on-going psychological need of this group in a separate manuscript, highlighting the importance of appropriate psychological input following transition.

Whilst medical advances have enabled serious IEI to be effectively cured with HSCT, the long-term sequelae of these procedures require adult immunology services experienced in managing long-term complications to provide our patients with the best quality of life possible.

## Supplementary Information

Below is the link to the electronic supplementary material.

Supplementary file1 (DOCX 63 KB)

## Data Availability

Not applicable.
