# Supplementary material for: Retrospective, Landmark Analysis of Long-term Adult Morbidity Following Allogeneic HSCT for Inborn Errors of Immunity in Infancy and Childhood
Source: J Clin Immunol. 2022 May 17;42(6):1230–43. doi: 10.1007/s10875-022-01278-6 (PMC9537214; doi:10.1007/s10875-022-01278-6)
Supplement: Supplementary file 1 — Supplementary file1 (DOCX 63 KB) [file 10875_2022_1278_MOESM1_ESM.docx]

| Patient number | Diagnosis | Event  1 | Time post HSCT | Event  2 | Time post HSCT | Event  3 | Time post HSCT | Event  4 | Time post HSCT | Event 5 | Time post HSCT | Event in last year of follow up |
| --- | --- | --- | --- | --- | --- | --- | --- | --- | --- | --- | --- | --- |
| 1 | JAK3 SCID | Intractable viral warts | 19 years | Recurrent genital herpes | 29 years | EBV viraemia | 38 years | NA | NA | NA | NA | Y – EBV viraemia/Intractable viral warts |
| 2 | ADA SCID | GBS | 6 years | Intractable viral warts | 18 years | Recurrent UTIs | 18 years | NA | NA | NA | NA | N |
| 3 | RAG2 Omenn | CD34 top-up | 14 years | Recurrent LRTIs | 27 years | Bronchiectasis | 27 years | Synthetic liver failure | 32 years | Death from MSOF | 33 years | Y – death from MSOF post-liver transplant |
| 4 | ADA SCID | T1DM | 24 years | NA | NA | NA | NA | NA | NA | NA | NA | Y – T1DM |
| 5 | ADA SCID | TB | 18 years | NA | NA | NA | NA | NA | NA | NA | NA | N |
| 6 | γ chain SCID | Intractable viral warts | 19 years | NA | NA | NA | NA | NA | NA | NA | NA | Y – Intractable viral warts |
| 7 | γ chain SCID | Intractable viral warts | 19 years | NA | NA | NA | NA | NA | NA | NA | NA | Y – Intractable viral warts |
| 8 | γ chain SCID | Persistent fungal nail infections | 19 years | NA | NA | NA | NA | NA | NA | NA | NA | Y –Fungal nail infection |
| 9 | γ chain SCID | Intractable viral warts | 13 years | NA | NA | NA | NA | NA | NA | NA | NA | Y – Intractable viral warts |
| 10 | γ chain SCID | Intractable viral warts | 8 years | NA | NA | NA | NA | NA | NA | NA | NA | Y – Intractable viral warts |
| 11 | γ chain SCID | Bronchiectasis | 5 years | NA | NA | NA | NA | NA | NA | NA | NA | Y – Bronchiectasis |
| 12 | γ chain SCID | Intractable viral warts | 15 years | NA | NA | NA | NA | NA | NA | NA | NA | Y – Intractable viral warts |
| 13 | T-B-NK+SCID | JIA | 3 years | CKD – stage 3a | 5 years | NA | NA | NA | NA | NA | NA | Y – CKD |
| 14 | γ chain SCID | Intractable viral warts | 5 years | NA | NA | NA | NA | NA | NA | NA | NA | Y – Intractable viral warts |
| 15 | γ chain SCID | Intractable viral warts | 12 years | CD34 top-up | 17 years | Moderate GvHD - skin and eyes | 18 years | SCC | 25 years | NA | NA | Y – Intractable viral warts |
| 16 | CD4 lymphopenia | EBV viraemia | 6 years | NA | NA | NA | NA | NA | NA | NA | NA | Y – EBV viraemia |
| 17 | Genetically undefined CID | Recurrent LRTIs | 15 years | NA | NA | NA | NA | NA | NA | NA | NA | Y – Recurrent LRTIs |
| 18 | WAS | Seronegative polyarthritis | 8 years | NA | NA | NA | NA | NA | NA | NA | NA | N |
| 19 | WAS | Bronchiectasis | 11 years | AIHA | 11 years | AIHA | 14 years | EBV viraemia | 15 years | NA | NA | Y - Bronchiectasis |
| 20 | XLP1 | Moderate GvHD | 5 years | NA | NA | NA | NA | NA | NA | NA | NA | N |
| 21 | CD40L | Recurrent LRTIs | 9 years | NA | NA | NA | NA | NA | NA | NA | NA | N |
| 22 | Chediak Higashi | Addison’s disease | 11 years | Recurrent RTI | 18 years | NA | NA | NA | NA | NA | NA | Y – Addison’s disease and recurrent URTI |
| 23 | X-CGD | CGD-related retinitis/uveitis | 12 years | NA | NA | NA | NA | NA | NA | NA | NA | N |
| 24 | X-CGD | Sudden cardiac death | 10 years |  |  |  |  |  |  |  |  | Y-Death |
| 25 | X-CGD | CGD-related discoid keratitis | 5 years | NA | NA | NA | NA | NA | NA | NA | NA | Y – Keratitis |
| 26 | X-CGD | Recurrent Bowen and SCC | 6 years | CKD – stage 3a | 7 years | NA | NA | NA | NA | NA | NA | Y - CKD |
| 27 | X-CGD | Bronchiolitis obliterans | 5 years | NA | NA | NA | NA | NA | NA | NA | NA | Y- Bronchiolitis obliterans |
| 28 | Genetically undefined SCID | Recurrent LRTIs | 5 years | Bronchiectasis | 10 years | NA | NA | NA | NA | NA | NA | Y - bronchiectasis and LRTIs |
| 29 | Genetically undefined SCID | JIA | 11 years | NA | NA | NA | NA | NA | NA | NA | NA | N |
| 30 | DOCK8 | Bronchiectasis | 7 years | NA | NA | NA | NA | NA | NA | NA | NA | Y – bronchiectasis |
| 31 | Undefined CID | Lymphedema | 5 years | Gastroenteritis with raised CRP and Hospital admission | 17 years | Pleural effusion requiring pleurodesis | 18 years | NA | NA | NA | NA | Y – Lymphedema and pleural effusion recollection |
| 32 | DOCK8 | Bacterial meningitis | 11 years | NA | NA | NA | NA | NA | NA | NA | NA | N |
| 33 | Genetically undefined CID | Transverse myelitis | 17 years | Bacterial pneumonia | 18 years | Orbital cellulitis | 19 years | NA | NA | NA | NA | Y – recurrent orbital cellulitis |
| 34 | RAG2 CID | Candida vocal cords | 17 years | NA | NA | NA | NA | NA | NA | NA | NA | N |
| 35 | PNP SCID | Bacterial pneumonia | 12 years | NA | NA | NA | NA | NA | NA | NA | NA | N |
| 36 | γ chain SCID | Intractable viral warts | 20 years | NA | NA | NA | NA | NA | NA | NA | NA | Y- Intractable warts |
| 37 | γ chain SCID | CD 34 top-up | 19 years | NA | NA | NA | NA | NA | NA | NA | NA | N |

**Supplementary Table 1. Details of Events.**

**Abbreviations:** JAK3 Janus kinase 3; SCID Severe combined immunodeficiency; EBV Epstein Barr Virus; ADA adenosine deaminase; GBS Guillain Barre Syndrome; UTIs urinary tract infections; RAG2 recombination activating 2 gene; LRTIs lower respiratory tract infections; MSOF multisystem organ failure; T1DM type 1 diabetes mellitus; Tb tuberculosis; CKD chronic kidney disease; GvHD graft versus host disease; SCC squamous cell carcinoma; CID combined immune deficiency; WAS Wiskott-Aldrich syndrome; AIHA autoimmune hemolytic anemia; XLP1 X-linked lymphoproliferative disease 1; CD40L CD40 Ligand deficiency; RTI respiratory tract infection; URTI upper respiratory tract infection; CGD chronic granulomatous disease; JIA juvenile idiopathic arthritis; DOCK8 Dedicator of cytokinesis 8 deficiency; CRP C reactive protein; PNP purine nucleoside phosphorylase; HSCT hematopoietic stem cell transplantation; NA not applicable; Y yes; N no.
